# *catena*-Poly[[μ_3_-hydroxido-tetra-μ_2_-pyrid­azine-1:2κ^4^ *N*:*N*′;1:3κ^2^ *N*:*N*′;2:3κ^2^ *N*:*N*′-tetrakis(selenocyanato)-1κ*N*,2κ*N*,3κ^2^ *N*-trizinc(II)]-μ-cyanido-1:2′κ^2^ *C*:*N*]

**DOI:** 10.1107/S1600536810029107

**Published:** 2010-07-24

**Authors:** Thorben Reinert, Jan Boeckmann, Inke Jess, Christian Näther

**Affiliations:** a Institut für Anorganische Chemie, Christian-Albrechts-Universität Kiel, Max-Eyth-Str. 2, 24098 Kiel, Germany

## Abstract

In the crystal structure of the title compound, [Zn_3_(NCSe)_4_(OH)(CN)(C_4_H_4_N_2_)_4_]_*n*_ one of the two crystallograph­ically independent zinc(II) cations is coordinated by two terminal *N*-bonded seleno­cyanato anions and two N atoms of two symmetry-related pyridazine ligands in a trigonal-bipyramidal geometry, while the other zinc(II) cation is coordinated by one terminal *N*-bonded seleno­cyanato anion, one μ-1,2-cyanido anion and three N atoms of three crystallographically independent pyridazine ligands in a slightly distorted octa­hedral coordination geometry. The zinc(II) atoms are further connected *via* a μ_3_-hydroxido anion into trinuclear building blocks. The formula unit consists of three zinc cations, four seleno­cyanato anions, one μ_3_-hydroxido anion, four pyridazine mol­ecules as well as one cyanido anion. The asymmetric unit contains half of a formula unit. One of the zinc atoms, two seleno­cyanato anions, two pyridazine ligands and the μ_3_-hydroxido anion are located on a crystallographic mirror plane, whereas the cyanido anion is located on a twofold rotation axis. Therefore, this anion is disordered due to symmetry. The cyanido anions connect the metal centres into polymeric zigzag chains propagating along the *a* axis.

## Related literature

For related μ_3_-hydroxo Zn coordination, see: Alexiou *et al.* (2005 ▶); Jana *et al.* (2006 ▶). For general background to inorganic–organic coordination polymers based on zinc(II) halides or pseudohalides and *N*-donor ligands, see: Näther *et al.* (2007 ▶); Bhosekar *et al.* (2006 ▶).
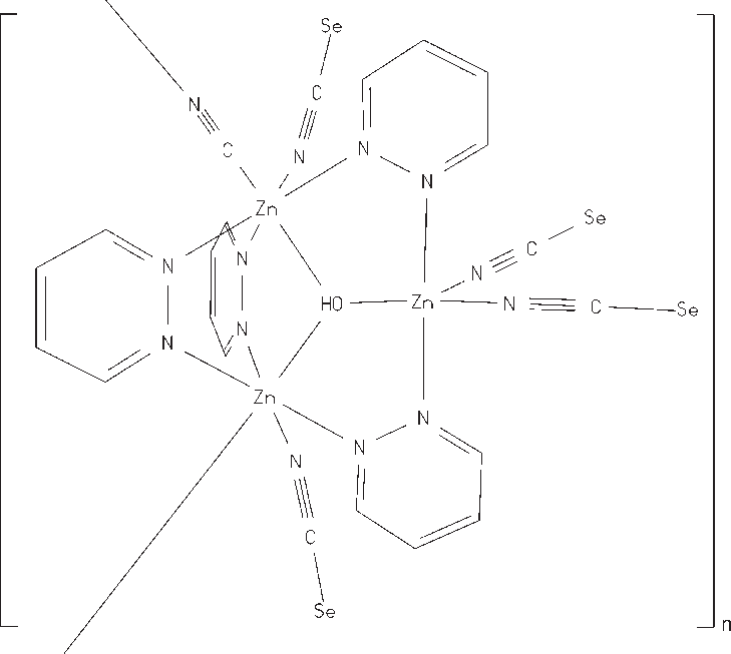


## Experimental

### 

#### Crystal data

[Zn_3_(CNSe)_4_(OH)(CN)(C_4_H_4_N_2_)_4_]
*M* *_r_* = 979.43
Orthorhombic, 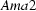

*a* = 15.6156 (12) Å
*b* = 22.6489 (16) Å
*c* = 8.6626 (5) Å
*V* = 3063.8 (4) Å^3^
*Z* = 4
Mo *K*α radiation
μ = 7.12 mm^−1^
*T* = 170 K
0.16 × 0.12 × 0.06 mm

#### Data collection

STOE IPDS-1 diffractometer
Absorption correction: numerical (*X-SHAPE* and *X-RED32*; Stoe, 2008 ▶) *T*_min_ = 0.196, *T*_max_ = 0.503
22283 measured reflections
3752 independent reflections
3588 reflections with *I* > 2σ(*I*)
*R*_int_ = 0.049

#### Refinement

*R*[*F* ^2^ > 2σ(*F* ^2^)] = 0.024
*wR*(*F* ^2^) = 0.058
*S* = 1.03
3752 reflections
206 parameters
1 restraint
H atoms treated by a mixture of independent and constrained refinement
Δρ_max_ = 0.42 e Å^−3^
Δρ_min_ = −0.65 e Å^−3^
Absolute structure: Flack (1983 ▶), 1747 Friedel pairs
Flack parameter: −0.012 (10)

### 

Data collection: *X-AREA* (Stoe, 2008 ▶); cell refinement: *X-AREA*; data reduction: *X-AREA*; program(s) used to solve structure: *SHELXS97* (Sheldrick, 2008 ▶); program(s) used to refine structure: *SHELXL97* (Sheldrick, 2008 ▶); molecular graphics: *XP* in *SHELXTL* (Sheldrick, 2008 ▶) and *DIAMOND* (Brandenburg, 2010 ▶); software used to prepare material for publication: *XCIF* in *SHELXTL*.

## Supplementary Material

Crystal structure: contains datablocks I, global. DOI: 10.1107/S1600536810029107/bt5302sup1.cif

Structure factors: contains datablocks I. DOI: 10.1107/S1600536810029107/bt5302Isup2.hkl

Additional supplementary materials:  crystallographic information; 3D view; checkCIF report

## Figures and Tables

**Table 1:** Selected bond lengths (Å)

Zn1—N1	1.985 (5)
Zn1—O1	1.997 (3)
Zn1—N2	2.015 (4)
Zn1—N11	2.214 (3)
Zn2—N3	2.092 (3)
Zn2—O1	2.1254 (19)
Zn2—N41	2.141 (3)
Zn2—N31	2.174 (3)
Zn2—N21	2.227 (3)
Zn2—N12	2.247 (3)

**Fig. 1.**
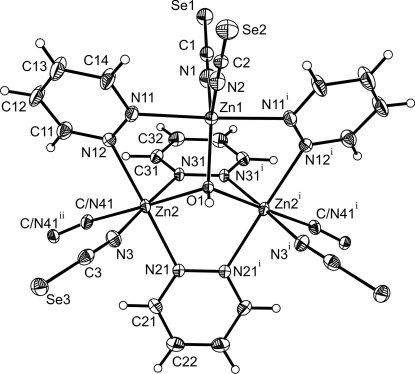
: Crystal structure of the discrete title compound with labelling and displacement ellipsoids drawn at the 50% probability level. Symmetry codes: i: -x + 3/2, y, z; ii: -x + 1, -y + 1, z.

**Fig. 2.**
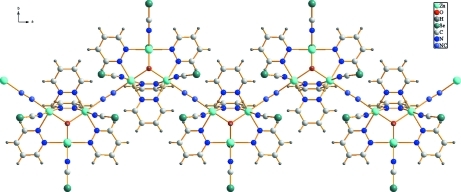
: Crystal structure of the title compound with view onto the polymeric chain along the crystallographic c-axis.

**Fig. 3.**
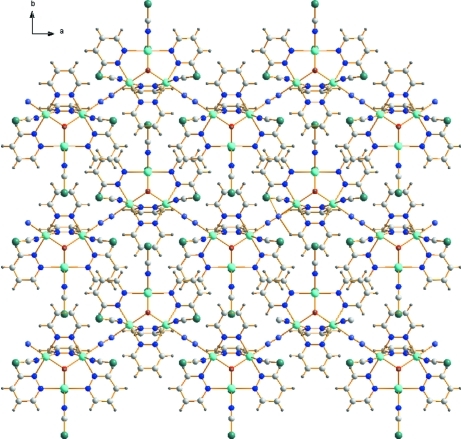
: Crystal structure of the title compound with topview onto the crystallographic ab-plane.

## supplementary crystallographic information

### Comment

Recently, we have investigated inorganic organic coordination polymers based on zinc(II) halides or pseudohalides and N-donor ligands (Näther *et al.*, 2007;  Bhosekar *et al.*,  2006). In our ongoing investigation on the synthesis, structures and properties of such compounds based on diamagnetic transition metals, pseudo-halides and N-donor ligands, we have reacted zinc(II) dinitrate, potassium selenocyanate and pyridazine in water. In this reaction single crystals were obtained by accident, which were identified as the title compound by single-crystal X-ray diffraction.

The title compound of composition [Zn_3_(NCSe)_4_(OH^-^)(CN^-^)(pyridazine)_4_]*_n_* (Fig. 1) represents a polymeric chain, in which trinuclear building units built up of three zinc(II) cations centered by a *µ*_3_-hydroxido anion are connected by µ-1,2-cyanido anions. One of the three zinc cations is coordinated by two selenocyanato anions, two N atoms of two pyridazine ligands and one *µ*_3_-hydroxido anion in a distorted trigonal bipyramidal coordination environment. The other two zinc(II) cations, are each coordinated by one selenocyanato, one *µ*-1,2-cyanido and one *µ*_3_-hydroxido anion and three N atoms of three pyridazine ligands in a slightly distorted octahedral coordination geometry. The Zn—N_pyridazine_ distances range between 2.174 (3) Å and 2.247 (3) Å, whereas the Zn—N_selenocyanato_ distances of the terminally N-bonded selenocyanato anions range between 1.985 (5) Å and 2.092 (3) Å. The angles around the trigonally bipyramidally coordinated metal centre range between 113.64 (18) - 130.90 (17)° and 177.39 (16)° (Tab. 1), whereas the angles around the octahedrally coordinated metal centres range between 83.09 (12) - 93.64 (12) and 178.62 (10)° (Tab. 1). The *µ*_3_-hydroxido anion coordination of the metal centres is not unusual and is similar to that found in related structures (Alexiou *et al.*, 2005; Jana *et al.*,  2006). The shortest Zn···Zn distances of the trinuclear metal centre amount to 3.4450 (5), whereas the shortest intrachain and interchain Zn···Zn distances amount to 5.2687 (5) and 9.0482 (6), respectively (Fig. 3).

### Experimental

Zn(NO_3_)_2_*x* 6H_2_O was obtained from Merck, KNCSe and pyridazine were obtained from Alfa Aesar. 1 mmol (128 mg) Zn(NO_3_)_2_*x* 6H_2_O, 2 mmol (288 mg) KNCSe, 2 mmol (160 mg) pyridazine and 3 ml water were reacted in a closed snap-vail without stirring. After the mixture has been standing for several days in the dark at room temperature light-yellow needle like single crystals of the title compound were obtained in a mixture with unknown phases.

### Refinement

The O—H hydrogen atom was located in difference map and was refined isotropically. The C—H H atoms were positioned with idealized geometry and were refined using a riding model with *U*_eq_(H) = 1.2 *U*_eq_(C) of the parent atom using C—H = 0.95 Å. Since there is only one atom of the cyanido anion in the asymmetric unit, this anion must be disordered over two equally occupied sites, C and N were refined sharing the same coordinates and the same displacement parameters.

### Crystal data

**Table:**

[Zn_3_(CNSe)_4_(OH)(CN)(C_4_H_4_N_2_)_4_]	*F*(000) = 1872
*M**_r_* = 979.43	*D*_x_ = 2.123 Mg m^−^^3^
Orthorhombic, *A**m**a*2	Mo *K*α radiation, λ = 0.71073 Å
Hall symbol: A 2 -2a	Cell parameters from 8001 reflections
*a* = 15.6156 (12) Å	θ = 2.5–28.1°
*b* = 22.6489 (16) Å	µ = 7.12 mm^−^^1^
*c* = 8.6626 (5) Å	*T* = 170 K
*V* = 3063.8 (4) Å^3^	Needle, light-yellow
*Z* = 4	0.16 × 0.12 × 0.06 mm

### Data collection

**Table:**

STOE IPDS-1 diffractometer	3752 independent reflections
Radiation source: fine-focus sealed tube	3588 reflections with *I* > 2σ(*I*)
graphite	*R*_int_ = 0.049
Phi scans	θ_max_ = 28.1°, θ_min_ = 2.5°
Absorption correction: numerical (*X-SHAPE* and *X-RED32* ;Stoe, 2008)	*h* = −20→20
*T*_min_ = 0.196, *T*_max_ = 0.503	*k* = −29→29
22283 measured reflections	*l* = −11→11

### Refinement

**Table:**

Refinement on *F*^2^	Hydrogen site location: inferred from neighbouring sites
Least-squares matrix: full	H atoms treated by a mixture of independent and constrained refinement
*R*[*F*^2^ > 2σ(*F*^2^)] = 0.024	*w* = 1/[σ^2^(*F*_o_^2^) + (0.0346*P*)^2^ + 4.6032*P*] where *P* = (*F*_o_^2^ + 2*F*_c_^2^)/3
*wR*(*F*^2^) = 0.058	(Δ/σ)_max_ = 0.002
*S* = 1.03	Δρ_max_ = 0.42 e Å^−^^3^
3752 reflections	Δρ_min_ = −0.65 e Å^−^^3^
206 parameters	Extinction correction: *SHELXL*, Fc^*^=kFc[1+0.001xFc^2^λ^3^/sin(2θ)]^-1/4^
1 restraint	Extinction coefficient: 0.00122 (10)
Primary atom site location: structure-invariant direct methods	Absolute structure: Flack (1983), 1747 Friedel pairs
Secondary atom site location: difference Fourier map	Flack parameter: −0.012 (10)

### Special details

**Table:**

Geometry. All e.s.d.'s (except the e.s.d. in the dihedral angle between two l.s. planes) are estimated using the full covariance matrix. The cell e.s.d.'s are taken into account individually in the estimation of e.s.d.'s in distances, angles and torsion angles; correlations between e.s.d.'s in cell parameters are only used when they are defined by crystal symmetry. An approximate (isotropic) treatment of cell e.s.d.'s is used for estimating e.s.d.'s involving l.s. planes.
Refinement. Refinement of *F*^2^ against ALL reflections. The weighted *R*-factor *wR* and goodness of fit *S* are based on *F*^2^, conventional *R*-factors *R* are based on *F*, with *F* set to zero for negative *F*^2^. The threshold expression of *F*^2^ > σ(*F*^2^) is used only for calculating *R*-factors(gt) *etc*. and is not relevant to the choice of reflections for refinement. *R*-factors based on *F*^2^ are statistically about twice as large as those based on *F*, and *R*- factors based on ALL data will be even larger.

### Fractional atomic coordinates and isotropic or equivalent isotropic displacement parameters (Å^2^)

**Table:**

	*x*	*y*	*z*	*U*_iso_*/*U*_eq_	Occ. (<1)
Zn1	0.7500	0.69593 (2)	0.31561 (6)	0.01454 (11)	
Zn2	0.63959 (2)	0.565314 (15)	0.35942 (4)	0.01401 (9)	
O1	0.7500	0.61613 (13)	0.4136 (4)	0.0124 (6)	
H1	0.7500	0.619 (4)	0.500 (12)	0.05 (3)*	
Se1	0.7500	0.70340 (2)	−0.25158 (6)	0.02447 (12)	
C1	0.7500	0.6962 (2)	−0.0473 (6)	0.0211 (10)	
N1	0.7500	0.6936 (2)	0.0866 (6)	0.0283 (10)	
Se2	0.7500	0.89017 (2)	0.57363 (7)	0.02695 (13)	
C2	0.7500	0.8214 (2)	0.4730 (6)	0.0180 (9)	
N2	0.7500	0.77724 (17)	0.4100 (5)	0.0209 (9)	
Se3	0.45849 (2)	0.591502 (15)	0.83059 (4)	0.02298 (9)	
C3	0.5344 (2)	0.58566 (15)	0.6754 (4)	0.0198 (7)	
N3	0.58164 (18)	0.58100 (13)	0.5733 (4)	0.0210 (6)	
N11	0.60825 (17)	0.69737 (12)	0.3112 (4)	0.0203 (6)	
N12	0.56988 (17)	0.64629 (12)	0.2776 (3)	0.0161 (5)	
C11	0.4879 (2)	0.64663 (16)	0.2356 (5)	0.0244 (7)	
H11	0.4619	0.6103	0.2074	0.029*	
C12	0.4386 (2)	0.69777 (17)	0.2312 (6)	0.0334 (10)	
H12	0.3799	0.6964	0.2026	0.040*	
C13	0.4770 (2)	0.74982 (17)	0.2690 (7)	0.0410 (12)	
H13	0.4463	0.7860	0.2689	0.049*	
C14	0.5641 (2)	0.74755 (16)	0.3081 (6)	0.0339 (10)	
H14	0.5927	0.7833	0.3334	0.041*	
N21	0.70660 (17)	0.48500 (12)	0.4454 (3)	0.0160 (5)	
C21	0.6652 (2)	0.43725 (16)	0.4926 (5)	0.0242 (7)	
H21	0.6043	0.4381	0.4938	0.029*	
C22	0.7066 (2)	0.38574 (17)	0.5405 (5)	0.0302 (8)	
H22	0.6751	0.3519	0.5720	0.036*	
N31	0.70651 (16)	0.55138 (11)	0.1423 (3)	0.0140 (5)	
C31	0.6645 (2)	0.54118 (15)	0.0115 (4)	0.0198 (6)	
H31	0.6037	0.5408	0.0133	0.024*	
C32	0.7064 (2)	0.53091 (16)	−0.1289 (4)	0.0222 (7)	
H32	0.6750	0.5242	−0.2211	0.027*	
N41	0.53181 (18)	0.51316 (13)	0.2907 (4)	0.0168 (6)	0.50
C41	0.53181 (18)	0.51316 (13)	0.2907 (4)	0.0168 (6)	0.50

### Atomic displacement parameters (Å^2^)

**Table:**

	*U*^11^	*U*^22^	*U*^33^	*U*^12^	*U*^13^	*U*^23^
Zn1	0.0139 (2)	0.0139 (2)	0.0157 (3)	0.000	0.000	0.00062 (18)
Zn2	0.00941 (14)	0.01595 (16)	0.01666 (19)	−0.00099 (12)	0.00125 (13)	−0.00029 (13)
O1	0.0098 (13)	0.0144 (14)	0.0130 (17)	0.000	0.000	0.0006 (12)
Se1	0.0252 (2)	0.0320 (3)	0.0162 (3)	0.000	0.000	−0.00013 (19)
C1	0.016 (2)	0.020 (2)	0.026 (3)	0.000	0.000	0.0012 (19)
N1	0.026 (2)	0.037 (3)	0.021 (3)	0.000	0.000	−0.0007 (19)
Se2	0.0326 (3)	0.0148 (2)	0.0334 (3)	0.000	0.000	−0.0050 (2)
C2	0.015 (2)	0.021 (2)	0.017 (2)	0.000	0.000	0.0075 (18)
N2	0.0187 (19)	0.0137 (18)	0.030 (2)	0.000	0.000	−0.0081 (17)
Se3	0.02313 (16)	0.02491 (16)	0.02089 (19)	0.00440 (12)	0.00737 (13)	0.00093 (13)
C3	0.0202 (16)	0.0189 (16)	0.0204 (19)	−0.0006 (12)	−0.0082 (13)	−0.0005 (12)
N3	0.0187 (13)	0.0253 (14)	0.0190 (15)	0.0008 (11)	0.0058 (11)	0.0006 (11)
N11	0.0139 (12)	0.0160 (12)	0.0309 (17)	0.0008 (9)	−0.0026 (11)	0.0011 (11)
N12	0.0154 (12)	0.0156 (12)	0.0172 (15)	−0.0007 (10)	−0.0005 (9)	0.0008 (10)
C11	0.0198 (16)	0.0213 (16)	0.032 (2)	−0.0004 (13)	−0.0060 (14)	−0.0014 (14)
C12	0.0176 (15)	0.0245 (19)	0.058 (3)	0.0035 (14)	−0.0155 (17)	0.0010 (18)
C13	0.0191 (18)	0.0224 (18)	0.081 (4)	0.0023 (14)	−0.018 (2)	−0.003 (2)
C14	0.0172 (16)	0.0157 (15)	0.069 (3)	0.0037 (12)	−0.0092 (17)	−0.0025 (17)
N21	0.0128 (13)	0.0180 (13)	0.0174 (16)	0.0004 (10)	0.0005 (10)	0.0028 (10)
C21	0.0179 (15)	0.0276 (18)	0.027 (2)	−0.0052 (13)	0.0014 (14)	0.0071 (14)
C22	0.0307 (19)	0.0229 (17)	0.037 (2)	−0.0041 (15)	−0.0013 (17)	0.0107 (16)
N31	0.0092 (12)	0.0165 (13)	0.0162 (14)	0.0003 (10)	0.0006 (10)	0.0003 (9)
C31	0.0162 (14)	0.0232 (16)	0.0199 (18)	0.0004 (12)	−0.0038 (13)	0.0023 (12)
C32	0.0266 (17)	0.0265 (16)	0.0133 (17)	−0.0002 (13)	−0.0041 (13)	−0.0006 (12)
N41	0.0131 (12)	0.0169 (13)	0.0203 (17)	0.0001 (10)	−0.0031 (11)	−0.0012 (11)
C41	0.0131 (12)	0.0169 (13)	0.0203 (17)	0.0001 (10)	−0.0031 (11)	−0.0012 (11)

### Geometric parameters (Å, °)

**Table:**

Zn1—N1	1.985 (5)	C11—C12	1.392 (5)
Zn1—O1	1.997 (3)	C11—H11	0.9500
Zn1—N2	2.015 (4)	C12—C13	1.363 (5)
Zn1—N11^i^	2.214 (3)	C12—H12	0.9500
Zn1—N11	2.214 (3)	C13—C14	1.402 (5)
Zn2—N3	2.092 (3)	C13—H13	0.9500
Zn2—O1	2.1254 (19)	C14—H14	0.9500
Zn2—N41	2.141 (3)	N21—C21	1.325 (4)
Zn2—N31	2.174 (3)	N21—N21^i^	1.355 (5)
Zn2—N21	2.227 (3)	C21—C22	1.398 (5)
Zn2—N12	2.247 (3)	C21—H21	0.9500
O1—Zn2^i^	2.1254 (19)	C22—C22^i^	1.355 (8)
O1—H1	0.75 (10)	C22—H22	0.9500
Se1—C1	1.777 (5)	N31—C31	1.330 (4)
C1—N1	1.161 (7)	N31—N31^i^	1.358 (5)
Se2—C2	1.784 (5)	C31—C32	1.400 (5)
C2—N2	1.140 (7)	C31—H31	0.9500
Se3—C3	1.797 (4)	C32—C32^i^	1.363 (7)
C3—N3	1.157 (5)	C32—H32	0.9500
N11—C14	1.330 (4)	N41—C41^ii^	1.159 (6)
N11—N12	1.335 (4)	N41—N41^ii^	1.159 (6)
N12—C11	1.331 (4)		
			
N1—Zn1—O1	113.64 (18)	C14—N11—Zn1	122.1 (2)
N1—Zn1—N2	115.5 (2)	N12—N11—Zn1	116.09 (19)
O1—Zn1—N2	130.90 (17)	C11—N12—N11	119.1 (3)
N1—Zn1—N11^i^	89.03 (9)	C11—N12—Zn2	123.8 (2)
O1—Zn1—N11^i^	91.19 (7)	N11—N12—Zn2	114.92 (19)
N2—Zn1—N11^i^	89.64 (7)	N12—C11—C12	123.0 (3)
N1—Zn1—N11	89.03 (9)	N12—C11—H11	118.5
O1—Zn1—N11	91.19 (7)	C12—C11—H11	118.5
N2—Zn1—N11	89.64 (7)	C13—C12—C11	118.0 (3)
N11^i^—Zn1—N11	177.39 (16)	C13—C12—H12	121.0
N3—Zn2—O1	93.64 (12)	C11—C12—H12	121.0
N3—Zn2—N41	89.99 (12)	C12—C13—C14	117.0 (3)
O1—Zn2—N41	176.37 (13)	C12—C13—H13	121.5
N3—Zn2—N31	176.69 (11)	C14—C13—H13	121.5
O1—Zn2—N31	83.09 (12)	N11—C14—C13	122.6 (3)
N41—Zn2—N31	93.28 (11)	N11—C14—H14	118.7
N3—Zn2—N21	92.62 (11)	C13—C14—H14	118.7
O1—Zn2—N21	89.28 (10)	C21—N21—N21^i^	119.2 (2)
N41—Zn2—N21	90.67 (11)	C21—N21—Zn2	122.7 (2)
N31—Zn2—N21	86.85 (10)	N21^i^—N21—Zn2	118.03 (7)
N3—Zn2—N12	86.05 (11)	N21—C21—C22	123.2 (3)
O1—Zn2—N12	91.18 (10)	N21—C21—H21	118.4
N41—Zn2—N12	88.96 (11)	C22—C21—H21	118.4
N31—Zn2—N12	94.50 (10)	C22^i^—C22—C21	117.6 (2)
N21—Zn2—N12	178.62 (10)	C22^i^—C22—H22	121.2
Zn1—O1—Zn2	113.35 (11)	C21—C22—H22	121.2
Zn1—O1—Zn2^i^	113.35 (11)	C31—N31—N31^i^	119.55 (18)
Zn2—O1—Zn2^i^	108.42 (14)	C31—N31—Zn2	121.7 (2)
Zn1—O1—H1	110 (6)	N31^i^—N31—Zn2	118.72 (7)
Zn2—O1—H1	106 (3)	N31—C31—C32	122.6 (3)
Zn2^i^—O1—H1	106 (3)	N31—C31—H31	118.7
N1—C1—Se1	177.7 (5)	C32—C31—H31	118.7
C1—N1—Zn1	175.6 (5)	C32^i^—C32—C31	117.84 (19)
N2—C2—Se2	179.4 (4)	C32^i^—C32—H32	121.1
C2—N2—Zn1	175.3 (4)	C31—C32—H32	121.1
N3—C3—Se3	178.2 (3)	C41^ii^—N41—N41^ii^	0.0 (4)
C3—N3—Zn2	165.6 (3)	C41^ii^—N41—Zn2	163.36 (12)
C14—N11—N12	120.3 (3)	N41^ii^—N41—Zn2	163.36 (12)

Symmetry codes: (i) −*x*+3/2, *y*, *z*; (ii) −*x*+1, −*y*+1, *z*.
